# Self-controlled wave propagation in hyperelastic media

**DOI:** 10.1038/s41598-017-08098-4

**Published:** 2017-08-08

**Authors:** Fengxian Xin, Tian Jian Lu

**Affiliations:** 1 State Key Laboratory for Strength and Vibration of Mechanical Structures, Xi’an Jiaotong University, Xi’an, 710049 P.R. China; 2 MOE Key Laboratory for Multifunctional Materials and Structures, Xi’an Jiaotong University, Xi’an, 710049 P.R. China

## Abstract

We demonstrate theoretically that an ultrasonic wave propagating in a hyperelastic medium can self-control its phase velocities. This phenomenon occurs because the propagation of the ultrasonic wave generates acoustic radiation stresses in the medium, which can induce large deformation of the medium with significant stiffening effect. In turn, such deformation reshapes the wave propagation while the deformation stiffening changes significantly the phase velocities of the wave till the acoustic radiation stresses are balanced by elastic stresses in the current configuration of the hyperelastic medium. As a result of deformation stiffening, an initially isotropic medium becomes anisotropic, thus enabling self-control or self-bending of the wave propagation. We further reveal that, due to snap-through instability of acoustomechanical deformation in the hyperelastic medium, the ultrasonic wave can discontinuously switch its phase velocities from one state to another by jumping over a large unstable regime. This self-control and switchable mechanism of ultrasonic wave propagation in homogenous hyperelastic media offers innovative design opportunities for phononic, thermal and acoustic materials and devices.

## Introduction

There is a long-lasting interest in controlling the propagation of acoustic/elastic waves in elastic media, for good understanding of such phenomenon is of paramount importance in a wide variety of engineering applications^1–19^, including waveguides, vibration and noise cancelling, acoustic cloaking, acoustic filters, ultrasonic imaging, etc. To realize effective control of wave propagation, several approaches have been employed, such as manipulating with artificial periodical microstructure^20^, material parameter transformation^21, 22^, active metamaterial design^23^, exerting pre-stress^24, 25^, harnessing deformation^26^ and mechanical instability^27, 28^. However, most of exiting approaches could not achieve instantaneous and revisable control of wave propagation, and they all highly relied on material/structure designs as well as external stimuli like mechanical or electrical loadings.

To circumvent these limitations, we propose a novel approach to accomplish instantaneous, revisable and self-controlled wave propagation in hyperelastic media such as soft materials (e.g., hydrogel). Upon harnessing acoustic radiation stresses induced by both wave propagation and material deformation stiffening, we demonstrate that the phase velocity of the wave can be controlled by its own magnitude. For illustration, we show that ultrasonic wave propagation in a thin layer of hyperelastic medium generates acoustic radiation stresses, which can not only deform the configuration of the layer but also stiffen its elastic stiffness. In turn, the changed configuration and altered stiffness reconstruct both the wave propagation and acoustic radiation stresses until a steady-state is achieved when the acoustic radiation stresses and the elastic stresses are balanced with each other. During this process, any alteration of the wave magnitude will lead to self-controlled change of deformation state in the medium, with corresponding alterations in deformation stiffening and phase velocity. We demonstrate further that the acoustomechanical deformation of hyperelastic medium exhibits snap-through instability, thus offering a new functionality of tunable and switchable control of wave propagation with discontinuously jumping phase velocities. Our work can inspire novel designs of phononic, thermal and acoustic materials/devices.

## Theoretical Analysis

**Figure 1:**
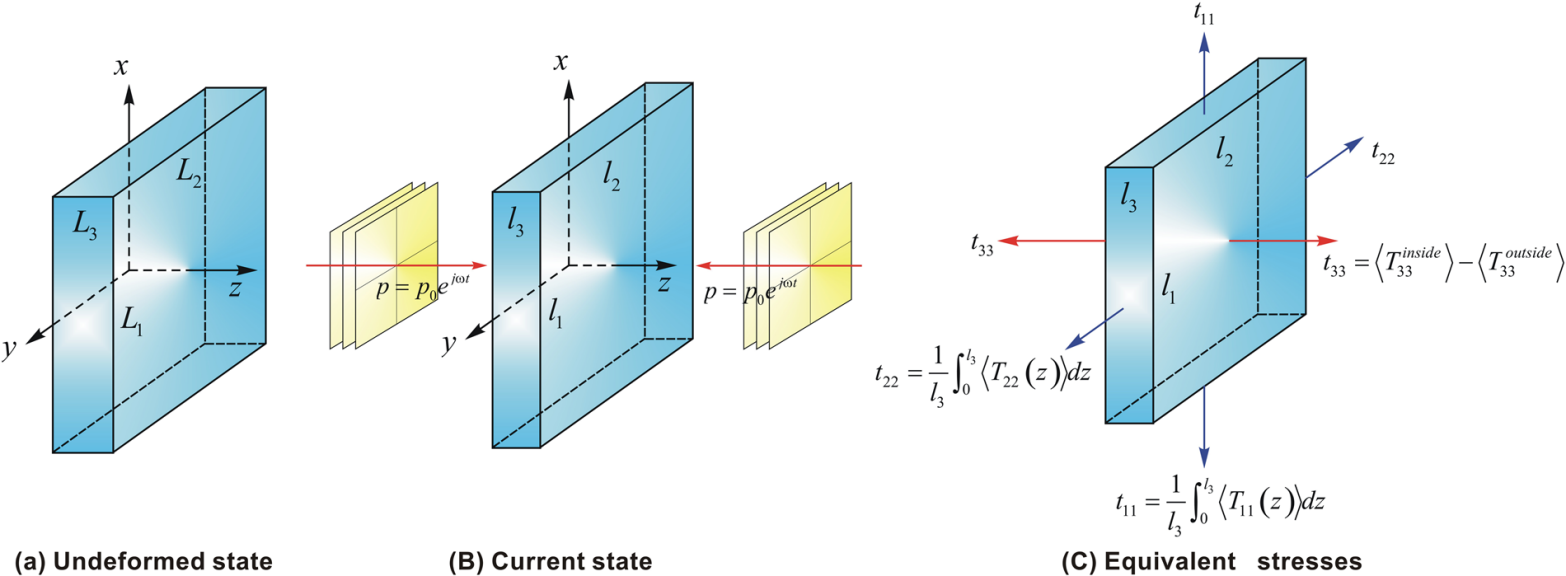
Deformation of a thin hyperelastic layer induced by acoustical radiation stresses. (**a**) In reference state, the layer has dimensions (*L*_1_, *L*_2_, *L*_3_). (**b**) In current state, the layer deforms to dimensions (*l*_1_, *l*_2_, *l*_3_) under two opposing sound pressure inputs *p*_*L*_ = *p*_0_ *e* ^*jωt*^ and *p*_*R*_ = *p*_0_ *e* ^*jωt*^ in the left and right sides of the medium, respectively. (**c**) Equivalent stresses induced by acoustic radiation stresses.

With reference to Fig. 1, consider a thin layer of hyperelastic medium (e.g., rubber and hydrogel), which is subjected to two opposing plane ultrasonic waves of high intensity and high frequency along its thickness direction. In the following, we will demonstrate theoretically that the ultrasonic waves propagating in the layer can self-control their phase velocities. That is, propagation of the two ultrasonic waves generates acoustic radiation stress, which are capable of inducing large deformation in the layer with significant stiffening: as a result, the ultrasonic waves can self-change their phase velocities.

As illustrated in Fig. 1, the media outside and inside the thin layer have acoustic impedance *ρ*_1_ *c*_1_ and *ρ*_2_ *c*_2_, respectively. The initial thickness of the layer (*e.g*, ~3 mm) is assumed to be one acoustic wavelength of Λ = 2*πc*/*ω* (Λ is the wavelength of ultrasonic wave in undeformed layer), while its in-plane dimensions (*e.g*., ~30 mm) are much larger. The two opposing waves with identical amplitude and frequency induce a standing-wave field in the thin layer. Due to acoustic momentum flux transfer between adjacent material particles, the propagation of ultrasonic waves in the hyperelastic medium can give rise to acoustic radiation forces. At sufficiently high frequencies, the hyperelastic medium bears negligible dynamical shear stress, thus behaving like a fluid in so far as ultrasonic wave propagation is of concern^29^. Correspondingly, the acoustic radiation force can be written in the form of a second-rank tensor^30–33^, as:

1 $$\langle {\bf{T}}\rangle =[\frac{\langle {p}^{2}\rangle }{2{\rho }_{a}{c}_{a}^{2}}-\frac{{\rho }_{a}\langle {\bf{u}}\cdot {\bf{u}}\rangle }{2}]{\bf{I}}+{\rho }_{a}\langle {\bf{u}}\otimes {\bf{u}}\rangle $$

where *p* is acoustic pressure, **u** is particle velocity vector, **I** is identity matrix, *ρ*_*a*_ and *c*_*a*_ are material density and acoustic speed, respectively, **T** is momentum flux tensor, and $\langle {\bf{T}}\rangle =(\omega /2\pi ){\int }_{0}^{2\pi /\omega }{\bf{T}}dt$, *ω* being angular frequency.

A focused ultrasonic wave can generate acoustic radiation forces that are sufficiently large to deform hyperelastic media. For example, a typical acoustic pressure of 1 MPa can generate an acoustic radiation stress of ~7 MPa in air. Therefore, ultrasonic wave propagation is capable of causing large deformation in hyperelastic media^29, 34, 35^. As shown in Fig. 1, the acoustic radiation stresses caused by the standing-wave field deform the thin layer hyperelastic from its initial state (*L*_1_, *L*_2_, *L*_3_) to the current state (*l*_1_, *l*_2_, *l*_3_) with principal stretches (*λ*_1_, *λ*_2_, *λ*_3_).

The deformation actuated by ultrasonic wave propagation can be characterized using the acoustomechanical model of hyperelastic media together with the nonlinear elasticity theory. The acoustomechanical Cauchy stress can be written as:

2 $${\boldsymbol{\sigma }}=\frac{{\bf{F}}}{J}\frac{\partial W({\bf{F}})}{\partial {\bf{F}}}-[\frac{\langle {p}^{2}\rangle }{2{\rho }_{a}{c}_{a}^{2}}-\frac{{\rho }_{a}\langle {\bf{u}}\cdot {\bf{u}}\rangle }{2}]{\bf{I}}-{\rho }_{a}\langle {\bf{u}}\otimes {\bf{u}}\rangle $$

where *W*(**F**) is the Helmholtz free energy function of hyperelastic medium, **F** = ∂**x**/∂**X** is the deformation gradient, **x** and **X** being the position vectors in reference and current configurations, respectively, and $J={\rm{\det }}({\bf{F}})$ is the Jacobian determinant of deformation gradient. Here, we adopt the compressible Gent model to describe the nonlinear elastic behavior of the hyperelastic medium. The Gent is capable of accounting for the effect of deformation stiffening in the medium^36, 37^, expressed as:

3 $$W({\bf{F}})=-\frac{\mu {J}_{m}}{2}\,\mathrm{ln}(1-\frac{{I}_{1}-3}{{J}_{m}})-\mu \,\mathrm{ln}\,J+(\frac{K}{2}-\frac{\mu }{3}-\frac{\mu }{{J}_{m}}){(J-1)}^{2}$$

where *μ* is the initial shear modulus, *K* is the initial bulk modulus, and *I*_1_ = tr(**B**) is the first invariant of the left Cauchy-Green tensor **B** = **F** · **F** ^**T**^. This model takes into account the material stiffening effect by introducing the polymer chain extension limit *J*_*m*_. When *J*_*m*_ → ∞, the Gent model degrades to the neo-Hookean model.

As seen from Eq. (2), the acoustomechanical Cauchy stress is contributed by both the elastic deformation stress and the acoustic radiation stress. In the absence of external mechanical force, deformation of the hyperelastic medium is actuated purely by ultrasonic waves. To describe the acoustic actuated deformation, let the acoustic radiation stresses balance with the elastic deformation stresses, as:

4 $${\bf{t}}=\frac{2}{J}\frac{\partial W}{\partial {I}_{1}}{\bf{B}}{\boldsymbol{+}}\frac{\partial W}{\partial J}{\bf{I}}=\frac{\mu {J}_{m}}{J({J}_{m}-{I}_{1}+3)}{\bf{B}}{\boldsymbol{+}}[(K-\frac{2\mu }{3}-\frac{2\mu }{{J}_{m}})(J-1)-\frac{\mu }{J}]{\bf{I}}$$

where the homogenized acoustic radiation stresses are given by:

5 $${t}_{1}=\frac{1}{{l}_{3}}{\int }_{0}^{{l}_{3}}\langle {T}_{11}(z)\rangle dz,{t}_{2}=\frac{1}{{l}_{3}}{\int }_{0}^{{l}_{3}}\langle {T}_{22}(z)\rangle dz,{t}_{3}=\langle {T}_{33}^{inside}({l}_{3})\rangle -\langle {T}_{33}^{outside}({l}_{3})\rangle $$

When subjected to acoustic radiation stresses, the thin hyperelastic layer of Fig. 1 can undergo large deformation with reduced in-plane area and expanded thickness, which will finally reach a stable state when the acoustic radiation stresses are balanced with the elastic stresses^29^. During the deformation process, the deformation stiffening effect of the hyperelastic medium steps in to change the phase velocities of the inside ultrasonic waves. In other words, during deformation, the two counter-propagating ultrasonic waves realize the self-controlling of their phase velocities via the gradually triggered stiffening effect by the waves themselves. Alternatively, from the viewpoint of a series of steady deformation states, changes in the amplitudes of input ultrasonic waves by acoustic sources can also alter the acoustic radiation stresses, the deformation and consequently the phase velocities of the waves. Broadly speaking, such control of phase velocities might be regarded as another self-controlled wave propagation, since it works by simply changing the amplitudes of the ultrasonic waves themselves: that is, apart from the input acoustic wave itself, other types of external stimuli are not needed to control the propagation of the wave.

Upon characterizing the acoustic triggered deformation, self-controlled wave propagation is demonstrated next. Here, we consider wave propagation in a deformed hyperelastic medium. Calculation of the acoustic radiation force is based on acoustic fields achieved in the hyperelastic medium as well as the surrounding medium. Firstly, the acoustic radiation force deforms the medium. Secondly, the deformed configuration of the medium reshapes the acoustic fields, generating a new acoustic radiation force until a balanced state is reached. Consequently, wave propagation in a deformed material can be considered as an incremental deformation superimposed upon existing deformation. In Eulerian coordinates, the incremental Cauchy stress $\tilde{{\boldsymbol{\sigma }}}$ is related to the displacement **u** = **u**(**x**, *t*) via the constitutive relation of the material, as:

6 $$\tilde{{\boldsymbol{\sigma }}}={\bf{C}}:\nabla {\bf{u}}$$

where ${\bf{C}}=\frac{1}{J}{\bf{F}}\frac{{\partial }^{2}W}{\partial {\bf{F}}\partial {\bf{F}}}{{\bf{F}}}^{{\bf{T}}}$ is the fourth-order incremental moduli tensor and “~” above a symbol denotes small increment in the quantity concerned. Wave propagation is then governed by the elastodynamical equation, as:

7 $$\nabla \cdot \tilde{{\boldsymbol{\sigma }}}=\rho \frac{{\partial }^{2}{\bf{u}}}{\partial {t}^{2}}$$

With *k* and **n** representing separately the wavenumber and propagation direction, the displacement is given by **u** = **U** *e* ^*j*(*k***n**·**x**−*ωt*)^. Incorporating Eqs (6) and (7), we obtain the Christoffel equation (**Q**(**n**) − *ρc* ^2^ **I**) · **u** = 0, where **Q**(**n**) = **C:n **⊗ **n** is the Christoffel tensor. If the compressible Gent model is adopted for the present hyperelastic medium, the Christoffel tensor can be expressed as^38^:

8 $${\bf{Q}}({\bf{n}})=(\tilde{\lambda }+{\tilde{\mu }}_{1}){\bf{n}}\otimes {\bf{n}}+{\tilde{\mu }}_{2}({\bf{n}}\cdot {\bf{B}}\cdot {\bf{n}}){\bf{I}}+{\tilde{\mu }}_{3}({\bf{n}}\cdot {\bf{B}})\otimes ({\bf{B}}\cdot {\bf{n}})$$

where

9 $$\begin{array}{rcl}\tilde{\lambda } & = & (2J-1)(K-\frac{2\mu }{3}-\frac{2\mu }{{J}_{m}})\\ {\tilde{\mu }}_{1} & = & (J-1)(K-\frac{2\mu }{3}-\frac{2\mu }{{J}_{m}})-\frac{\mu }{J}\\ {\tilde{\mu }}_{2} & = & \frac{\mu {J}_{m}}{J({J}_{m}-{I}_{1}+3)}\\ {\tilde{\mu }}_{3} & = & \frac{2\mu {J}_{m}}{J{({J}_{m}-{I}_{1}+3)}^{2}}\end{array}$$

Under acoustic inputs, the hyperelastic medium deforms to current state with stiffened tangential stiffness, and the Christoffel equation determines the three polarized phase velocities along prescribed propagation direction. The three phase velocities correspond to purely-transversal wave, quasi-transversal wave, and quasi-longitudinal wave, respectively. The phase velocity of the purely-transversal wave is expressed as:

10 $${c}_{pt}=\sqrt{J{\tilde{\mu }}_{2}({\bf{n}}\cdot {\bf{B}}\cdot {\bf{n}})/{\rho }_{0}}$$

while that of the quasi-longitudinal wave is:

11 $${c}_{ql}=\sqrt{J[\tilde{\lambda }+{\tilde{\mu }}_{1}+{\tilde{\mu }}_{2}({\bf{n}}\cdot {\bf{B}}\cdot {\bf{n}})+{\tilde{\mu }}_{3}({\bf{n}}\cdot {\bf{B}})\otimes ({\bf{B}}\cdot {\bf{n}})]/{\rho }_{0}}$$

The phase velocity of the quasi-transversal wave can be numerically calculated by solving the Christoffel equation. It should be pointed out that the three phase velocities actually describe the anisotropic stiffness property of the medium at deformed state, and do not mean the existence of the corresponding waves in the medium. For the case considered herein, only the quasi-longitudinal wave exists since the input ultrasonic wave is taken as a compressional wave propagating in the dynamical fluid-like medium. Note that while the hyperelastic medium behaves like a fluid for high frequency wave propagation, it can still propagate low frequency shear waves. Therefore, the phase velocity of two shear waves is only meaningful for low frequency shear waves, while Eq. (11) is meaningful for both low frequency and high frequency longitudinal waves. At certain deformed state of the medium, to a large extent, the phase speed of input compressive acoustic wave in the medium is approximately equal to that of the quasi-longitudinal wave, because the second-order and higher order terms of the wave are much smaller than its linear components. Consequently, the phase velocity of quasi-longitudinal wave is adopted in the current study to demonstrate the speed of self-controlled waves in hyperelastic media. For completeness, the two other phase velocities for low frequency transverse waves are also presented although they do not really exist in the cases considered. Because the compressible Gent model has accounted for the effect of deformation stiffening, the acoustomechanical deformation of the hyperelastic material can remarkably alter the three phase velocities, as elucidated below.

## Results and Discussion

To clearly show the self-controlled propagation of acoustic waves in a hyperelastic medium, we theoretically calculate the phase velocities and the corresponding slowness of wave propagation. Detailed theoretical formulations can be referred to our recent works^29, 34, 35, 39–41^, and hence are omitted here. Most polymer materials are relatively soft and nearly incompressible, and hence can be described by the compressible Gent model. In the present study, relevant material parameters (initial shear modulus *μ* = 4.6 kPa, Poisson ratio *ν* = 0.49 and extension limit *J*_*m*_ = 3) are selected from the range where they usually belong to.

**Figure 2:**
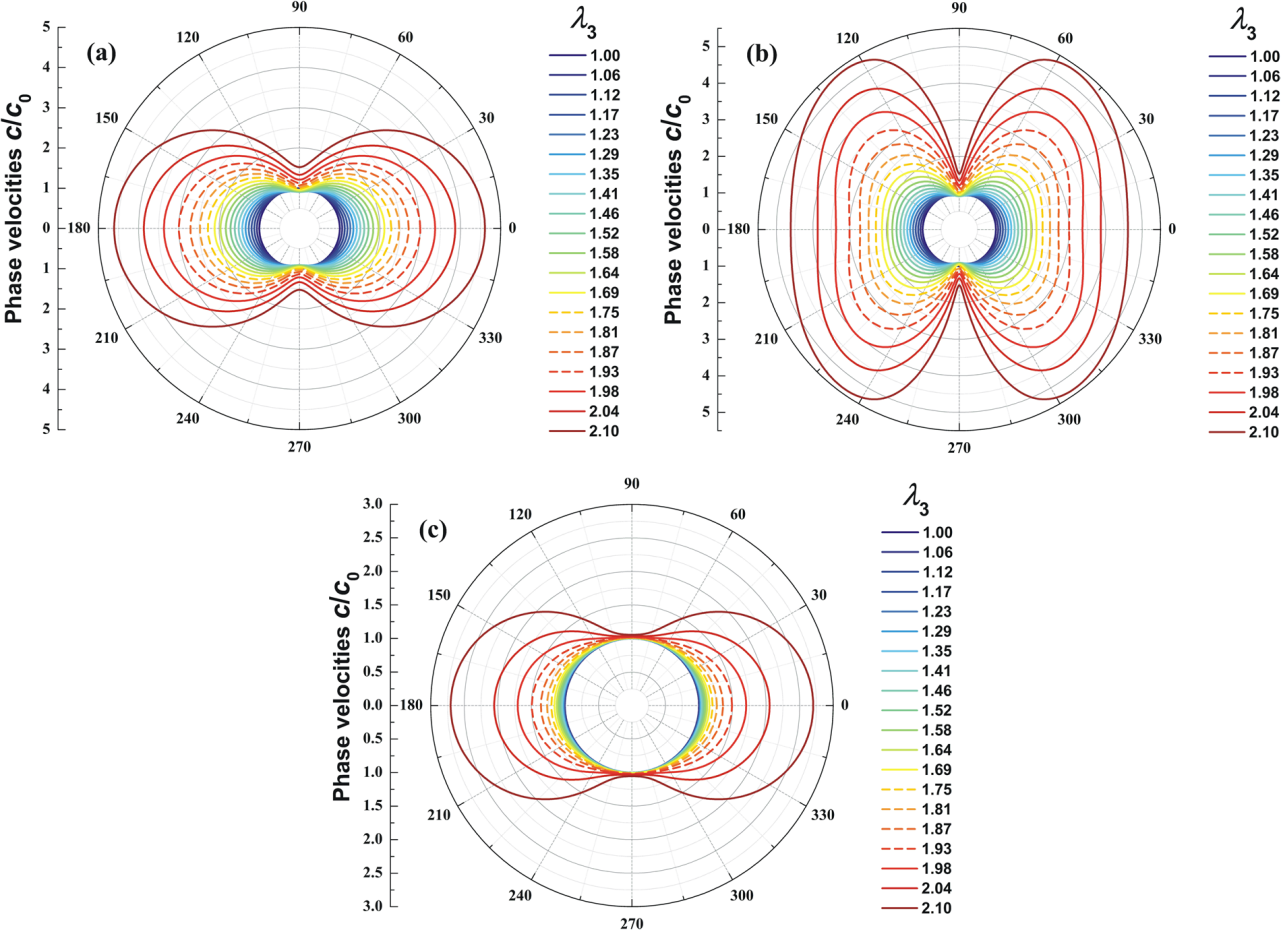
Phase velocity polar diagrams of self-controlled elastic waves in a compressible hyperelastic medium in the *yz* plane: (**a**) purely-transversal velocity; (**b**) quasi-transversal velocity; (**c**) quasi-longitudinal velocity. Solid lines correspond to stable regime while dashed lines denote unstable regime.

Figure 2 presents the polar diagrams of phase velocities (as functions of out-of-plane stretch) in the *yz* plane (Fig. 1), with the horizontal axis representing the *z*-axis and the vertical axis representing the *y*-axis. The three polarized phase velocities include the purely-transversal velocity, the quasi-transversal velocity and the quasi-longitudinal velocity, as shown in Fig. 2(a–c), which are normalized by their respective values at undeformed state. The results of Fig. 2 demonstrate that acoustic-triggered deformation is able to significantly stiffen the material and thereby change the polarized phase velocities. Such deformation is directly related to acoustic input, as will be shown later. In other words, the acoustic input is able to self-control its phase velocities by deforming and stiffening the material lying along its propagation path. Specifically, wave propagation in the thickness direction not only gives rise to a large stretch in this direction but also stiffens the material in this direction, thus causing significantly increased phase velocity in the *z*-direction and slightly increased phase velocity in the *y*-direction (Fig. 2). At the undeformed state (i.e., zero acoustic input, *λ*_3_ = 1), all the three polarized phase velocities are isotropic, as represented by the three innermost circles in Fig. 2. With increasing acoustic input and deformation, the polarized phase velocities become anisotropic, as evidenced by the butterfly-type diagrams shown in Fig. 2. Note that, as the acoustomechanical deformation of a hyperelastic medium is inevitably associated with snap-through instability, the stable profiles in Fig. 2 are plotted using solid lines while the unstable profiles are plotted with dashed lines.

For completeness, the slowness surfaces of elastic waves are investigated as well, since the group velocities of the corresponding elastic waves can be graphically derived from the slowness plots^42^. In other words, the plots of slowness surface can give intuitive perception of the group velocities of elastic waves in any given polarized direction. The slowness surface of an elastic wave is defined as the locus of **s** = *c* ^−1^ **n**, with **n** varying over unit vectors. Therefore, the purely-transversal and quasi-longitudinal waves have the following slowness:

12 $${s}_{pt}=\sqrt{{\rho }_{0}/J{\tilde{\mu }}_{2}({\bf{n}}\cdot {\bf{B}}\cdot {\bf{n}})}$$

13 $${s}_{ql}=\sqrt{{\rho }_{0}/J[\tilde{\lambda }+{\tilde{\mu }}_{1}+{\tilde{\mu }}_{2}({\bf{n}}\cdot {\bf{B}}\cdot {\bf{n}})+{\tilde{\mu }}_{3}({\bf{n}}\cdot {\bf{B}})\otimes ({\bf{B}}\cdot {\bf{n}})]}$$

**Figure 3:**
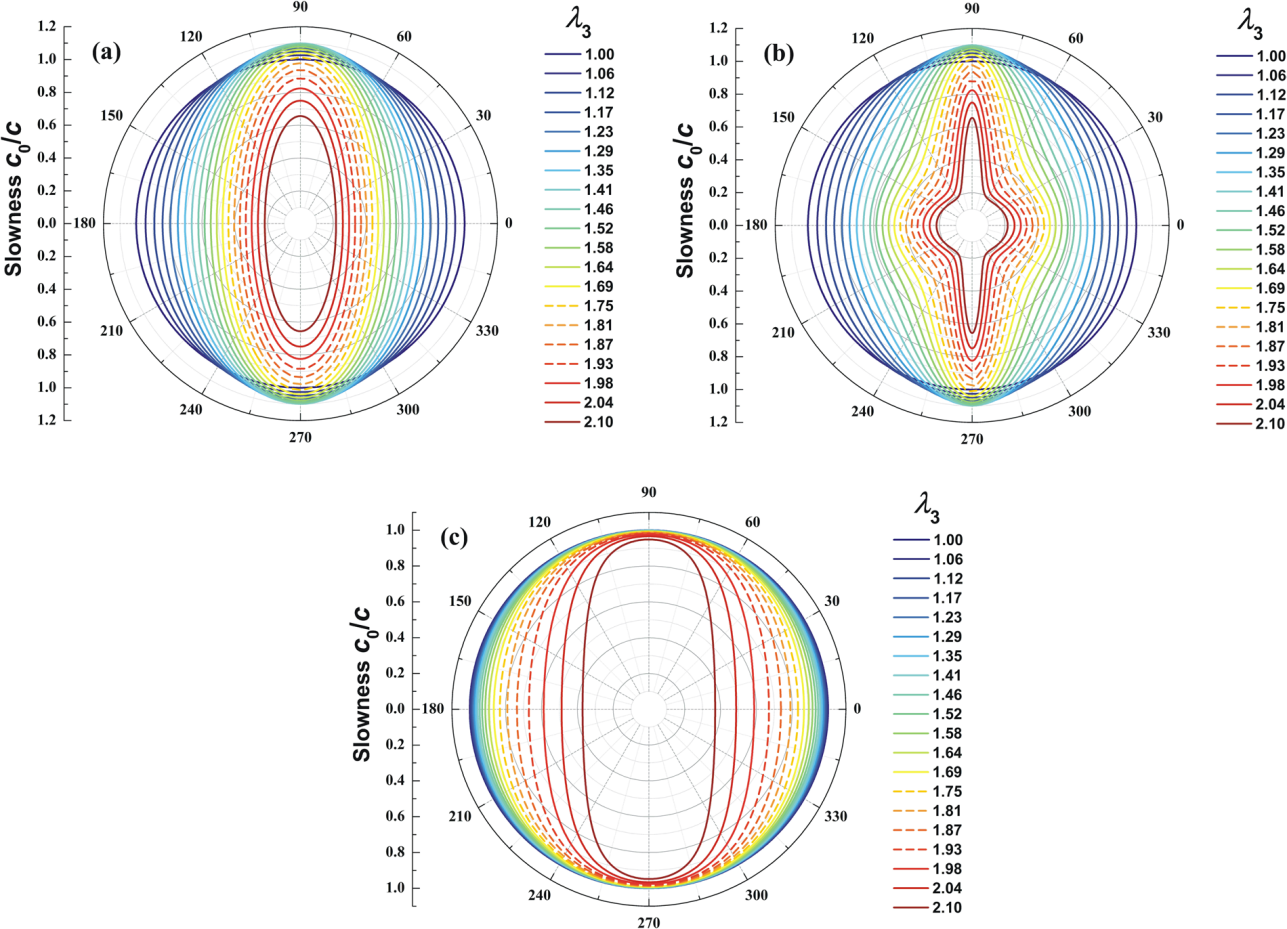
Slowness surface polar diagrams for self-controlled elastic waves in a compressible hyperelastic medium in *yz* plane: (**a**) purely-transversal velocity; (**b**) quasi-transversal velocity; (**c**) quasi-longitudinal velocity. Solid and dashed lines denote stable and unstable regimes, respectively.

The quasi-transversal wave slowness has been numerically calculated, which can be graphically illustrated to reveal the properties of wave propagation under prescribed acoustic inputs. Figure 3 presents the slowness surface polar diagrams for the polarized wave modes of the acoustomechanically deforming hyperelastic medium in *yz* plane (*i.e*., horizontal axis is the *z*-axis and vertical axis is the *y*-axis). As a result of the snap-through instability of acoustomechanical deformation, solid and dashed lines are used to denote stable and unstable states, respectively. When the thin hyperelastic layer is stretched along its thickness direction, deformation stiffening remarkably shifts the slowness diagram profiles in this direction (Fig. 3). The larger the acoustic inputs are, the more significantly the slowness surface changes. Therefore, we have demonstrated that the phase velocities and slowness of wave propagation in an initially isotropic hyperelastic medium can be controlled by the wave itself via acoustic radiation force and deformation stiffening. If one further considers obliquely incident acoustic inputs, it is expected to realize self-bending or programmable propagation of acoustic waves in a homogenous and isotropic medium that can be deformation stiffened, by simply changing the magnitude(s) of the acoustic inputs.

In practice, the phase velocities and the corresponding slowness surfaces of elastic waves in the hyperelastic medium can be measured by applying the time-difference method. For the considered case of Fig. 1, a thin sheet of hyperelastic medium is subjected to two counterpropagating ultrasonic waves of high intensity and high frequency. The sheet is largely deformed and stiffened by the generated acoustic radiation stress, which in turn alters the phase velocities and slowness surfaces of the elastic waves. To measure the phase velocities, one needs to send another weak idler impulse wave through the thin sheet. The distance *L* of the weak idler impulse wave passing through and the time difference *t* between the sending and the receiving waves can thence be detected. The corresponding phase velocity can be calculated as *c* = *L*/*t* and the slowness is *s* = *t*/*L*. As a matter of fact, the application of weak idler in experimental measurement is similar to techniques used in nonlinear optics^43^.

**Figure 4:**
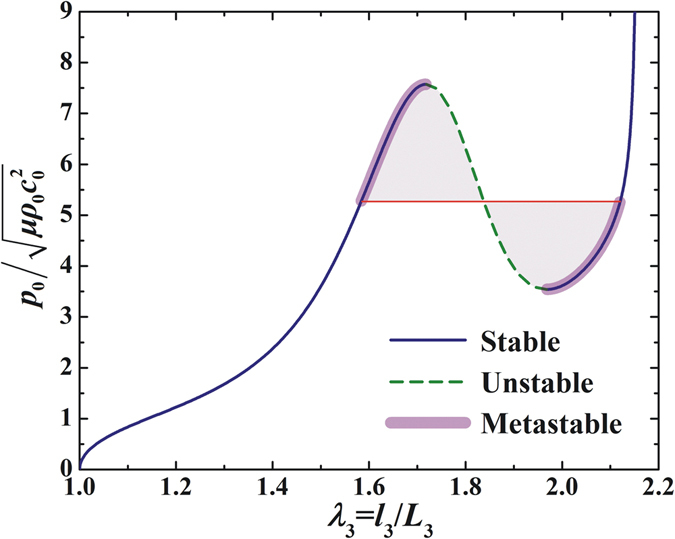
The acoustical load-stretch relationship exhibits snap-through instability with stable, unstable and metastable regimes (dashed line means unstable region). The metastable region is identified by applying the Maxwell rule.

To highlight the effect of deformation stiffening, Fig. 4 plots the normalized acoustic input ${p}_{0}/\sqrt{\mu {\rho }_{0}{c}_{0}^{2}}$ as a function of the out-of-plane stretch. Normalization of the acoustic input stems from the formulation of acoustic radiation stress in Eq. (1). Because the acoustical radiation stress scales as ${p}_{0}^{2}/({\rho }_{a}{c}_{a}^{2})$ (if one notes that the first term in Eq. (1) is $\langle {p}^{2}\rangle /(2{\rho }_{a}{c}_{a}^{2})$), the non-dimensional acoustic radiation stress scales as ${p}_{0}^{2}/(\mu {\rho }_{a}{c}_{a}^{2})$, *p*_0_ being the amplitude of input sound pressure and *μ* the initial shear modulus of the medium. Therefore, the acoustic input can be normalized as ${p}_{0}/\sqrt{\mu {\rho }_{0}{c}_{0}^{2}}$ by taking the square root of the normalized acoustic radiation stress ${p}_{0}^{2}/(\mu {\rho }_{a}{c}_{a}^{2})$.

From Fig. 4 it can be seen that, as the stretch increases, the normalized acoustic input exhibits strong stiffening effect: it firstly rises to a peak value, then goes down, and then increases again. This phenomenon is attributed to the snap-through instability associated with acoustomechanical deformation, i.e., the deformation jumps directly from the peak to another large deformation state over an unstable region. Consequently, by applying the Maxwell rule for phase transition, the acoustic load-stretch curve of Fig. 4 can be divided into three distinct regimes: stable, metastable and unstable regimes.

**Figure 5:**
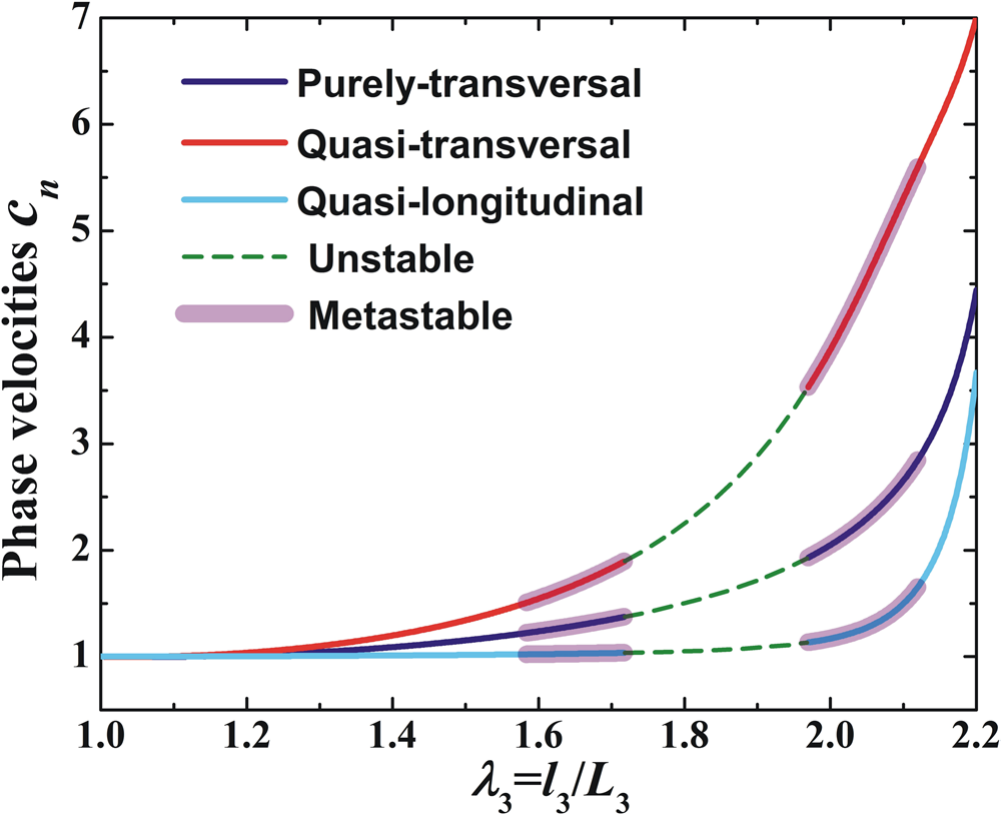
Normalized phase velocities of purely-transversal, quasi-transversal and quasi-longitudinal waves plotted as functions of out-of-plane stretch in the direction $${\bf{n}}=[\begin{array}{ccc}0 & \sqrt{3}/2 & 1/2\end{array}]$$. Dashed lines represent unstable region, and metastable region is identified by applying the Maxwell rule.

**Figure 6:**
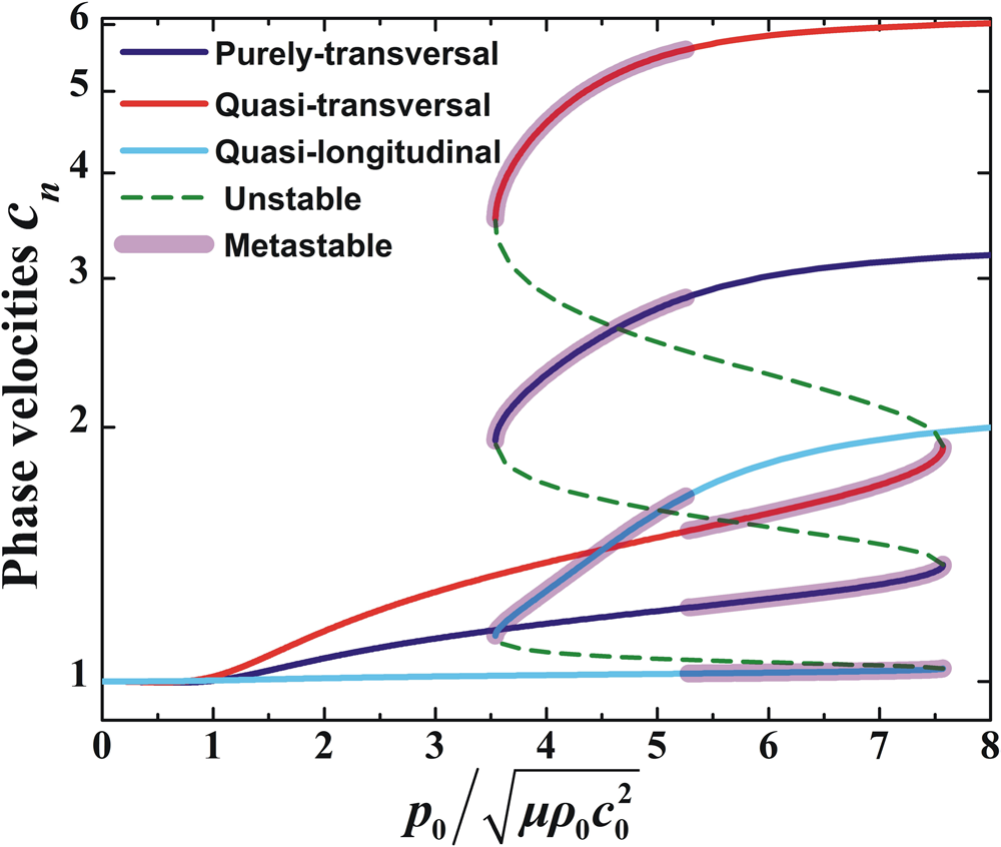
Normalized phase velocities of purely-transversal, quasi-transversal and quasi-longitudinal waves plotted as functions of acoustic input in the direction $${\bf{n}}=[\begin{array}{ccc}0 & \sqrt{3}/2 & 1/2\end{array}]$$. Dashed lines represent unstable region, and metastable region is identified by applying the Maxwell rule.

The variation trends of the normalized phase velocities with stretch and acoustic inputs in the direction $${\bf{n}}=[\begin{array}{ccc}0 & \sqrt{3}/2 & 1/2\end{array}]$$ are presented in Figs 5 and 6, respectively. Due to deformation stiffening, the polarized phase velocities noticeably increase with incresing stretch or acoustic inputs. Given that the acoustomechanical deformation possesses snap-through instability, the alteration of phase velocities can also be divided into stable, metastable and unstable regimes. Therefore, by harnessing the snap-through instability, the wave is capable of controlling its phase velocities to achieve discontinuous switch from a small value to another much larger value. Particularly, deformation stiffening significantly affects the quasi-transversal wave phase velocity, secondly the purely-transversal wave phase velocity, and thirdly the quasi-longitudinal wave phase velocity. Correspondingly, by harnessing the instability, the switch of the quasi-transversal wave phase velocity is the largest, then the purely-transversal wave phase velocity, followed by the quasi-longitudinal wave phase velocity.

## Conclusions

We demonstrate theoretically the novel mechanism of self-controlled ultrasonic wave propagation in hyperelastic media by applying the acoustic radiation force generated by the wave itself. Self-controlled propagation occurs during the deformation process via the gradually triggered material stiffening by the ultrasonic waves themselves. Harnessing the snap-through instability associated with the acoustomechanical deformation of hyperelastic media, we show that a large switch of wave propagation with jumping phase velocity can be realized, accompanied with jumping material deformation. We further reveal that, under acoustic loading, the initially isotropic hyperelastic medium can not only undergo large deformation but also become anisotropic. In turn, the deformed configuration and stiffened material stiffness of hyperelastic medium reshapes the acoustic fields. The complicate interplay between wave propagation and material deformation reaches a stable state when the wave-generated acoustic radiation forces are balanced with the deformation-generated elastic stresses. This balanced state is determined by the magnitude of input acoustic wave and therefore can be controlled by the input wave itself. It is also expected that acoustic radiation forces generated by oblique incident acoustic waves are capable of inducing shear deformation and shear stiffening effect, causing in turn the waves to self-bend their propagation routes. The proposed mechanism of self-controlled wave propagation based on acoustic radiation forces and material stiffening effects offers new avenues for designing advanced photonic, thermal, mechanical and acoustic materials/devices.
